# The neutrophil-to-lymphocyte ratio: A potential predictor of poor prognosis in adult patients with trauma and traumatic brain injury

**DOI:** 10.3389/fsurg.2022.917172

**Published:** 2022-08-23

**Authors:** Jinghong Xu, Shuhe Li, Ka Yin Lui, Xiaodong Song, Xiaoguang Hu, Lu Cao, Yanping Zhu, Fa Huang, Xiaobin Lin, Changjie Cai

**Affiliations:** ^1^ Department of Critical Care Medicine, The First Affiliated Hospital of Sun Yat-Sen University, Guangzhou, China; ^2^ Department of Extracorporeal Circulation, The First Affiliated Hospital of Sun Yat-Sen University, Guangzhou, China; ^3^ Department of Pharmacology, The First Affiliated Hospital of Sun Yat-Sen University, Guangzhou, China; ^4^ Department of Anesthesiology, Guangzhou Women and Children's Medical Center, Guangzhou, China

**Keywords:** neutrophil to lymphocyte ratio, trauma, traumatic brain injury, intensive care unit mortality, intensive care unit stay

## Abstract

**Purpose:**

This study aimed to determine the prognostic impact of the neutrophil-to-lymphocyte ratio (NLR) in critically ill trauma patients.

**Methods:**

This retrospective study involved adult trauma patients from 335 intensive care units (ICUs) at 208 hospitals stored in the eICU database. The primary outcome was ICU mortality. The lengths of ICU and hospital stay were calculated as the secondary outcomes. The multivariable logistic regression model was used to identify independent predictors of mortality. To identify the effect of the NLR on survival, a 15-day survival curve was used.

**Results:**

A total of 3,865 eligible subjects were enrolled in the study. Univariate analysis showed that patients in the group with a higher NLR were more likely to receive aggressive methods of care delivery: mechanical ventilation, vasopressor, and antibiotics ( *P* < 0.001 for all). The ICU, in-hospital, and 15-day mortality rates of the four groups increased in turn (*P* < 0.001 for all). The multivariable logistic Cox regression model indicated that a higher NLR was an independent risk factor of ICU mortality in trauma patients. ROC analysis showed that the NLR had better predictive capacity on the mortality of patients with traumatic brain injury (TBI) than those with trauma (AUC 0.725 vs. 0.681). An NLR > 7.44 was an independent risk factor for ICU death in patients with TBI (OR: 1.837, 95% CI: 1.045–3.229) and TBI victims whose NLR > 7.44 had a 15-day survival disadvantage (*P* = 0.005).

**Conclusion:**

A high NLR is associated with a poor prognosis in trauma patients, even worse in patients with TBI. An NLR > 7.44 is an independent risk factor for death in patients with TBI.

## Introduction

Trauma is the injury of human tissues or organs caused by mechanical injury factors, which causes millions of deaths worldwide every year (1). Traumatic brain injury (TBI) is the leading cause of mortality in patients aged <40 years (2). The numerous complications in patients who survive the injury, such as hospital-acquired infection, injury-related dementia, and hemiplegia, lead to an economic burden on society and impact the quality of life of individuals and their families (3). Subsequent pathological processes caused by trauma, such as acute infection, hemorrhage, and severe tissue injury, all lead to an increase in neutrophils. Palmer et al. pointed out that the increased neutrophils contribute to vascular dysfunction in the very early hours of the insult, which can directly lead to the destruction of the blood–brain barrier (BBB) and affect the prognosis of patients with trauma (4). The lymphocyte count usually reflects the host's immune defenses against an external attack. Patients with insufficient number of circulating lymphocytes could experience difficulties in starting the innate immune response, leaving the body helpless in the face of trauma. Many studies have tried to combine neutrophils with lymphocytes to comprehensively assess the conditions of patients. The neutrophil-to-lymphocyte ratio (NLR), as an objective, available, low-cost, and reproducible indicator of inflammation, has attracted the attention of medical providers and has been used to evaluate the severity and prognosis of pancreatitis, infectious diseases, and cardiovascular diseases (5–7). The high level of the NLR often indicates a poor prognosis for these diseases. In recent years, many research studies have begun to follow with interest the prognostic value of the NLR in trauma patients. In a retrospective study, Evren et al. mentioned that an NLR on the 5th day after admission greater than or equal to 7.92 can be used as a marker for increased in-hospital mortality (8). A single-center study in Asia also showed that NLR levels were associated with 6-month mortality in patients with TBI (9). We conducted this retrospective multicenter study to explore the relationship between NLR value within 24 h of entering intensive care unit (ICU) and clinical outcomes of trauma, aiming to provide more insights for early intervention and prognosis of trauma patients.

## Materials and methods

### Data resource

Study data were retrospectively extracted from the eICU Collaborative Research Database, which is a large multicenter critical care database made available by Philips Healthcare in partnership with the MIT Laboratory for Computational Physiology (eicu-crd.mit.edu). Our access to the database was approved after completion of the NIH web-based training course called “Protecting Human Research Participants” (Certification Number: 2093226). There are over 200,000 admissions in 335 ICUs at 208 hospitals located across the United States between the years 2014 and 2015 stored in the eICU database (10).

### Study population and data extraction

**Figure 1:**
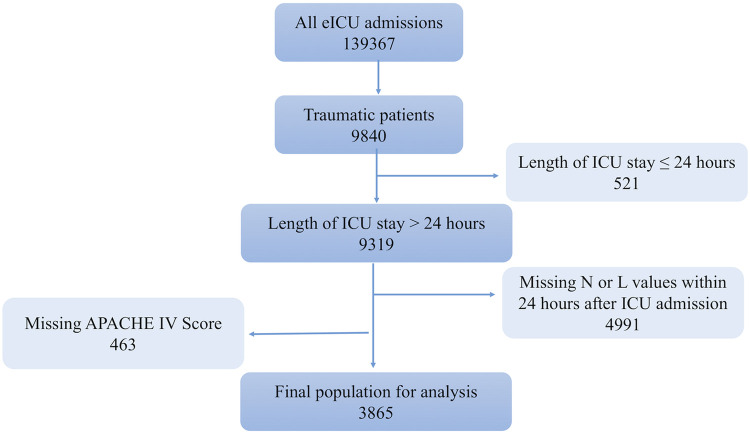
Flowchart of study cohort selection.

All patients older than 18 years with a diagnosis of trauma were included in the study. The data extraction process is shown in Figure 1. Patients were excluded for missing neutrophil or lymphocyte count data during the first 24 h of ICU stay. Patient information, including demographics, comorbidities, vital signs, and laboratory tests over the first 24 h, was extracted for analysis. The true age of patients over 89 years old was obscured in the database, and we used their median age of 91.5 as the surrogate age. The neutrophil-to-lymphocyte ratio was calculated by dividing the absolute neutrophil count by the absolute lymphocyte count. Acute Physiology and Chronic Health Evaluation (APACHE IV), Glasgow Coma Scale (GCS), treatments (use of vasopressor and antibiotics and use of mechanical ventilation and dialysis) and discharge status, and duration of ICU and hospital stay were abstracted for this study. The primary outcomes in our study were the ICU mortality, 15-day mortality, hospital, and ICU length-of-stay (LOS) as indirect measures for resource utilization as secondary outcomes.

Data extraction was performed using structure query language (SQL) with PostgreSQL tools (version 9.6; PostgreSQL Global Development Group).

### Statistical analysis

Data of normal distribution were expressed as mean and standard deviation (SD) and compared using the t-test or one-way ANOVA across groups. However, skewed data were described as median and interquartile range and compared by using the Kruskal–Wallis (multiple groups) or Wilcoxon rank-sum test (two groups). Categorical variables were expressed as numbers and percentages and compared by using Pearson's chi-square test or Fisher's exact test as appropriate. We divided patients into four groups according to the NLR quartile and compared all the study variables across groups by using univariate analysis. The multivariable logistic regression analysis was conducted to determine whether the NLR was independently associated with ICU mortality.

Receiver operating characteristic (ROC) curve analysis (11) was conducted to assess the predictive capacity of the NLR on the mortality of patients with trauma or TBI. TBI patients were divided into two groups according to the optimum cutoff value. Next, univariate analysis was performed to evaluate the effect of the NLR on the primary and secondary outcomes of patients with TBI. The multivariate logistic regression analysis was also conducted to assess the independent risk factors of ICU mortality for TBI patients.

Statistical analyses were performed using the software SPSS (version 22.0) or R (version 3.5.1). A two-sided *P* < 0.05 was considered statistically significant.

## Results

### The study of traumatic patients

#### Population and baseline characteristics

After removing those patients missing neutrophil or lymphocyte counts, a total of 3,865 trauma patients were included in our study and divided into four groups, named groups 1, 2, 3, and 4, according to the interquartile interval of the NLR (Table 1).

**Table 1:** Univariate analysis of baseline characteristics in 3,865 trauma patients by NLR category.

	Group 1	Group 2	Group 3	Group 4	*P*
	<4.38 (966)	4.38–7.55 (972)	7.55–13.00 (960)	>13.00 (967)	
NLR time (h)	10.33 ± 6.107	9.93 ± 6.570	8.99 ± 6.457	7.66 ± 6.235	<0.001
Sex, *n* (%)					<0.001
Female	441 (45.7)	348 (35.8)	361 (37.7)	396 (41.0)	
Male	525 (54.3)	624 (64.2)	597 (62.3)	571 (59.0)	
Age, *n* (%)					0.054
<45	239 (24.7)	255 (26.2)	255 (26.6)	233 (24.1)	0.546
45–64	307 (31.8)	259 (26.6)	262 (27.3)	245 (25.3)	0.010
65–89	374 (38.7)	398 (40.9)	382 (39.8)	426 (44.1)	0.095
>90	46 (4.8)	60 (6.2)	61 (6.4)	63 (6.5)	0.340
Ethnicity, *n* (%)					0.059
Native American	6 (0.6)	2 (0.2)	10 (1.0)	4 (0.4)	0.089
African American	79 (8.2)	66 (6.8)	71 (7.4)	58 (5.4)	0.098
Asian	12 (1.2)	12 (1.2)	6 (0.6)	10 (1.0)	0.501
Caucasian	755 (78.2)	788 (81.1)	762 (79.4)	824 (85.2)	<0.001
Hispanic	58 (6.0)	56 (5.8)	56 (5.8)	38 (3.9)	0.145
Other/Unknown	45 (4.7)	38 (3.9)	43 (4.5)	34 (3.5)	0.570
Region *n* (%)					0.220
Midwest	349 (36.1)	378 (38.9)	356 (37.1)	382 (39.5)	0.388
Northeast	90 (9.3)	105 (10.8)	102 (10.6)	131 (13.5)	0.026
South	196 (20.3)	202 (20.8)	209 (21.8)	185 (19.1)	0.544
West	295 (30.5)	252 (25.9)	272 (28.3)	245 (25.3)	0.041
Other/Unknown	36 (3.7)	35 (3.6)	21 (2.2)	24 (2.5)	0.110
TBI, *n* (%)	509 (52.7)	449 (46.2)	434 (45.2)	406 (42.0)	<0.001
ICU type, *n* (%)					<0.001
Med-Surg ICU	638(66.0)	613 (63.1)	634 (66.0)	633 (65.5)	0.461
MICU	22 (2.3)	21 (2.2)	29 (3.0)	38 (3.9)	0.072
SICU	66 (6.8)	75 (7.7)	97 (10.1)	82 (8.5)	0.062
CCU/CTICU	39 (4.0)	76 (7.8)	62 (6.5)	80 (8.3)	0.001
Neuro ICU	201 (20.8)	187 (19.2)	138 (14.4)	134 (13.9)	<0.001
BMI, *n* (%)					0.218
<18.5	30 (3.3)	42 (4.6)	36 (3.9)	52 (5.6)	0.079
18.5–24.9	353 (38.8)	320 (34.8)	335(36.7)	359 (38.8)	0.210
25.0–29.9	289 (31.8)	280 (30.5)	272(29.8)	259 (28.0)	0.492
30.0–34.9	133 (14.6)	168 (18.3)	171 (18.7)	143 (15.5)	0.042
35.0–39.9	61 (6.7)	60 (6.5)	55 (6.0)	63 (6.8)	0.907
≥40.0	44 (4.8)	49 (5.3)	44 (4.8)	49 (5.3)	0.919
Surgery, *n* (%)	482 (49.9)	512 (52.7)	517 (53.9)	488 (50.5)	0.261
Comorbidities, *n* (%)					
CKD	50 (5.2)	53 (5.5)	48 (5.0)	66 (6.8)	0.289
COPD	28 (2.9)	37 (3.8)	38 (4.0)	53 (5.5)	0.035
Heart failure	18 (1.9)	27 (2.8)	31(3.2)	47 (4.9)	0.002
Cancer	7 (0.7)	7 (0.7)	20 (2.1)	29 (3.0)	<0.001
Hypertension	128 (13.3)	117 (12.0)	130 (13.5)	123 (12.7)	0.770
Diabetes	88 (9.1)	77 (7.9)	69 (7.2)	72 (7.4)	0.410
MODS, *n* (%)	160 (16.6)	267 (27.5)	308 (32.1)	386 (39.9)	<0.001
MAP < 60 mmHg, *n* (%)	40 (4.1)	71 (7.3)	77 (8.0)	116 (12.0)	<0.001
APACHE IV	42 (31–54)	46 (34.25–62)	52 (37–67)	59 (43–79)	<0.001
Sedation score	−0.48 ± 1.477	−0.94 ± 1.856	−1.23 ± 1.974	−1.18 ± 2.042	<0.001
Analgesic score	4.08 ± 3.699	3.53 ± 3.721	3.20 ± 3.467	3.52 ± 3.585	0.043

BMI, body mass index; ICU, intensive care unit; MICU, medical ICU; Med-Surg ICU, medical-surgical ICU; SICU, surgical ICU; CCU, cardiac care unit; CTICU, cardiothoracic ICU; CKD, chronic kidney disease; COPD, chronic obstructive pulmonary disease; AIDS, acquired immune deficiency syndrome; MAP, mean arterial pressure; GCS, Glasgow Coma Scale; APACHE IV, Acute Physiology and Chronic Health Evaluationn.

There were slightly more males than females in the trauma population, in line with our knowledge that males are more likely to experience physical trauma (12). In 208 hospitals in the United States, over 60% of trauma patients received treatment in Med-Surg ICU. Patients in group 4 were more likely to suffer from heart failure, cancer, and chronic obstructive pulmonary disease (COPD). There seemed to be a certain correlation between the severity of trauma patients entering the ICU and the NLR. Among populations with trauma, patients with a higher NLR were more likely to develop MODS. A total of 1,121 traumatic patients developed MODS, accounting for 16.6%, 27.5%, 32.1%, and 39.9% of groups 1, 2, 3, and 4, respectively. Compared with that in group 4, the APACHE IV score was lower in groups 1, 2, and 3. What is more, more people with MAP less than 60 mmHg were found in group 4. The APACHE IV score was also higher in the group with a high NLR.

#### Univariate analysis of clinical outcomes

It is clear in Table 2 that patients in the group with the higher NLR were more likely to receive aggressive methods of care delivery. The number of patients receiving mechanical ventilation and antibiotics increased progressively among the four groups (*P *< 0.001 for both). The incidence of vasopressor usage was significantly lower in groups 1 and 2 compared with that in group 4 (*P *< 0.001). The ICU, hospitalization, and 15-day mortality rates increased gradually among the four groups. The hospital mortality rate in group 1 was 2.3%, but it was as high as 17.5% in group 4. The length of ICU stay and hospitalization, as an indirect measure for resource utilization, also showed an upward trend among the four groups (*P *< 0.001 for both). The median of hospital stay started at 4.42 days in group 1, prolonged gradually with an increased NLR, and reached 8.13 days in group 4.

**Table 2:** Univariate analysis of clinical outcomes in 3,865 patients by NLR category.

	Group 1	Group 2	Group 3	Group 4	*P*
	<4.38 (966)	4.38–7.55 (972)	7.55–13.00 (960)	>13.00 (967)	
Ventilation *n* (%)	173 (17.9)^a^	255 (26.2)^b^	347 (36.1)^c^	430 (44.5)^d^	<0.001
Dialysis *n* (%)	13 (1.3)	20 (2.1)	18 (1.9)	28 (2.9)	0.112
Vasopressor *n* (%)	42 (4.3)^a^	63 (6.5)^a^	96 (10.0)^b^	154 (15.9)^c^	<0.001
Antibiotics *n* (%)	99 (10.2)^a^	141 (14.5)^b^	200 (20.8)^c^	259 (26.8)^d^	<0.001
ICU LOS (days)	1.71 (0.96–2.96)	2.13 (1.25–3.95)	2.71 (1.46–5.88)	3.29 (1.63–6.83)	<0.001
Hospital LOS (days)	4.42 (2.46–7.67)	5.92 (3.54–10.00)	7.02 (4.13–12.86)	8.13 (4.71–14.13)	<0.001
ICU-free LOS (days)	2.04 (0.91–4.46)	3.08 (1.13–5.83)	3.29 (1.00–6.50)	3.42 (1.08–7.25)	<0.001
ICU mortality, *n* (%)	17 (1.8)^a^	37 (3.8)^b^	59 (6.1)^c^	117 (12.1)^d^	<0.001
15-day mortality, *n* (%)	22 (2.3)^a^	57 (5.9)^b^	84 (8.8)^b^	155 (16.0)^c^	<0.001
In-Hospital mortality, *n* (%)	22 (2.3)^a^	60 (6.2)^b^	89 (9.3)^b^	169 (17.5)^c^	<0.001

LOS, length of stay.

The *P*-value of the two groups marked with the same letter was greater than the adjusted *P*-value, and there was no statistical difference; otherwise, the *P*-value of the two groups marked with the different letter is smaller than the adjusted *P*-value, and there was statistical difference.

#### Multivariate regression model and adjusted survival curves

**Figure 2:**
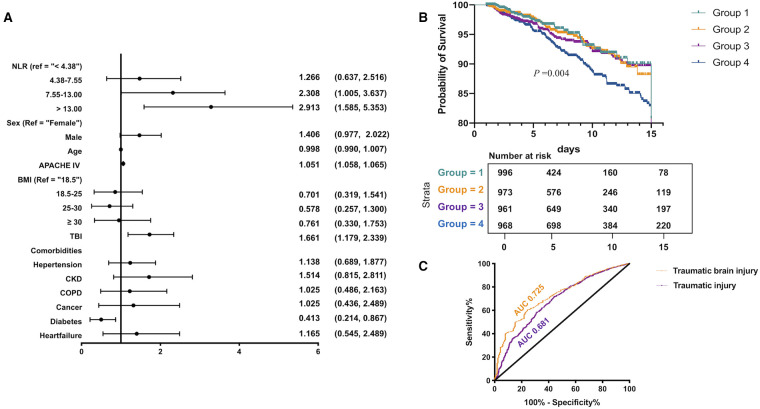
Multivariate regression model and adjusted survival curves in traumatic patients: (**A**) multivariate logistic regression model was used to show that the higher NLR was an independent risk factor of ICU mortality for traumatic patients, (**B**) the adjusted cumulative survival curve indicated the marked survival disadvantage in group 4 compared with that in group 1 (*P* = 0.004), and (**C**) ROC curve analysis indicated that the NLR in predicting ICU mortality in patients with TBI was higher than that in patients with trauma injury.

Taking into consideration the significant differences in the characteristic baseline among the four groups, the multivariate logistic regression model was used to find out if the NLR was an independent risk factor of ICU mortality. After univariate analysis (data not given), the appropriate variables were included in the regression model (Figure 2A). After adjusting covariates listed in the model, the NLR was independently associated with the ICU mortality of trauma victims. The risk of mortality in groups 2, 3, and 4 was 1.266, 2.308, and 2.913 times higher than that in group 1 (*P* = 0.502, *P *= 0.048, *P *< 0.001, respectively). The adjusted cumulative survival curve is shown in Figure 2B, indicating the marked survival disadvantage in group 4 compared with that in group 1 (*P* = 0.004).

#### ROC curve analysis on trauma patients and TBI patients

According to Figure 2A, patients with traumatic brain injury are an independent risk factor for high ICU mortality. It has great guiding significance for clinical diagnosis and treatment to study the risk factors affecting the clinical prognosis of TBI patients. Therefore, we explored the relationship between the NLR and the clinical outcomes of patients with TBI. We validated the capacity of the NLR in predicting the hospital death of trauma patients and TBI patients (Figure 2C). The area under the ROC curve of trauma patients was 0.681, and the optimal cutoff value was 9.75, corresponding to a sensitivity and specificity of 65.8% and 65.3%. While for victims with TBI, the maximum area under the ROC curve was 0.725, and the optimal cutoff value was 7.439, the sensitivity and specificity could reach up to 77.7% and 60.2%, respectively. This indicated that the value of the NLR in predicting ICU mortality in patients with TBI was higher than that in patients with trauma injury. Hence, we focused on the relationship between the NLR and the clinical prognosis of patients with TBI. The relationship between the first NLR value within ICU 72 hours and clinical outcome was shown in Supplementary table 1–2.

### The study of patients with traumatic brain injury

#### Univariate analysis of baseline characteristics and clinical outcomes

A total of 1,798 adult patients with traumatic brain injury who had lymphocyte and neutrophil counts detected within 24 h after entering ICU were divided into two groups according to the optimal cutoff in the ROC analysis (Table 3). Patients in the high NLR group were more likely to develop a GCS score of less than 8 (*P* < 0.001). The APACHE IV score (GCS excluded) of the high NLR group was significantly higher than that of the low NLR group (46.0 vs. 32.0, *P* < 0.001).

**Table 3:** Univariate analysis of baseline characteristics and main outcomes in 1,798 patients with TBI.

	Low	High	*P*
	NLR ≤ 7.44 (934)	NLR > 7.44 (864)	
NLR time (h)	10.14 ± 6.323	8.47 ± 6.348	<0.0001
Sex, *n* (%)			0.032
Female	376 (40.3)	305 (35.3)	
Male	558 (59.7)	559 (64.7)	
Age, *n* (%)			0.008
<45	222 (23.8)	266 (30.7)	0.001
45–64	260 (27.8)	227 (26.3)	0.456
65–89	410 (43.9)	332 (38.4)	0.019
>90	42 (4.5)	39 (4.5)	0.986
BMI, *n* (%)			0.760
<18.5	31 (3.3)	33 (3.8)	0.567
18.5–24.9	344 (36.8)	321 (37.2)	0.888
25.0–29.9	292 (31.3)	249 (28.8)	0.259
30.0–34.9	132 (14.1)	136 (15.7)	0.339
35.0–39.9	49 (5.2)	53 (6.1)	0.416
≥40.0	39 (4.2)	35 (4.1)	0.894
Ethnicity			0.745
African American	76 (8.1)	60 (6.9)	0.339
Asian	10 (1.1)	9 (1.0)	0.952
Caucasian	746 (79.9)	691 (80.0)	0.956
Hispanic	52 (5.6)	46 (5.3)	0.820
Native American	5 (0.5)	10 (1.2)	0.147
Other/unknown	45 (4.8)	48 (5.6)	0.557
ICU type, *n* (%)			0.002
Med-Surg ICU	567 (60.7)	558 (64.6)	0.090
MICU	14 (1.5)	19 (2.2)	0.269
SICU	58 (6.2)	74 (8.6)	0.056
CCU/CTICU	30 (3.2)	33 (3.8)	0.484
Neuro ICU, *n* (%)	265 (28.4)	180 (20.8)	<0.0001
Comorbidities, *n* (%)			
CKD	40 (4.3)	38 (4.4)	0.908
COPD	27 (2.4)	20 (2.3)	1.000
Heart failure	20 (2.1)	17 (2.3)	0.615
Cancer	4 (0.4)	16 (1.9)	0.006
Hypertension	125 (13.4)	117 (13.5)	0.945
Diabetes	66 (7.1)	53 (6.1)	0.449
Hematoma location, *n* (%)			
Epidural hematoma	181 (17.2)	160 (21.5)	0.022
Subdural hematoma	202 (19.2)	181 (24.3)	0.009
Subarachnoid hemorrhage	126 (12.0)	73 (9.8)	0.152
Intracerebral hematoma	334 (31.7)	197 (26.5)	0.016
Not known	210 (19.9)	133 (17.9)	0.272
Surgery, *n* (%)	614 (58.3)	457 (61.4)	0.289
MODS *n* (%)	205 (21.9)	319 (36.9)	<0.001
MAP < 60 mmHg, *n* (%)	48 (24.2)	98 (31.2)	0.108
GCS ≤ 8, *n* (%)	174 (22.4)	332 (44.0)	<0.001
APACHE IV (GCS excluded)	32.00 (20.00–47.00)	46.00 (27.00–70.00)	<0.001
Sedation score	−0.82 ± 1.801	−1.61 ± 2.165	<0.001
Analgesic score	3.80 ± 3.691	2.34 ± 3.041	<0.001
Ventilation, *n* (%)	209 (22.4)	387 (44.8)	<0.001
Dialysis, *n* (%)	10 (1.1)	15 (1.7)	0.314
Vasopressor *n* (%)	37 (4.0)	112 (13.0)	<0.001
Antibiotics *n* (%)	80 (8.6)	173 (20.0)	<0.001
ICU LOS (days)	1.88 (1.08–3.67)	3.54 (1.71–7.87)	<0.001
Hospital LOS (days)	4.81 (2.67–8.61)	7.52 (3.92–13.91)	<0.001
ICU-free LOS (days)	2.13 (0.87–4.87)	2.71 (0.29, 6.03)	0.160
ICU mortality, *n* (%)	32 (3.4)	97 (11.2)	<0.001
15-day mortality, *n* (%)	45 (4.8)	96 (11.1)	<0.001
In-hospital mortality, *n* (%)	42 (4.5)	143 (16.6)	<0.001

BMI, body mass index; ICU, intensive care unit; MICU, medical ICU; Med-Surg ICU, medical-surgical ICU, SICU, surgical ICU; CCU, cardiac care unit; CTICU, cardiothoracic ICU; CKD, chronic kidney disease; COPD, chronic obstructive pulmonary disease; AIDS, acquired immune deficiency syndrome; MAP, mean arterial pressure; GCS, Glasgow Coma Scale; APACHE IV, Acute Physiology and Chronic Health Evaluation; LOS, length of stay; TBI, traumatic brain injury.

**Figure 3:**
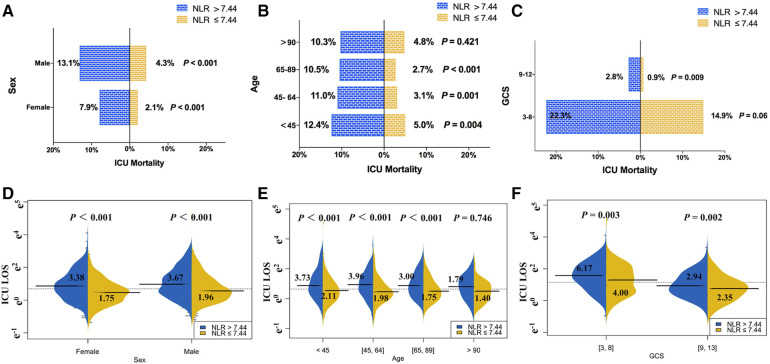
Comparison of ICU mortality and the length of ICU stay between TBI patients with NLR ≤ 7.44 and NLR > 7.44 in different subgroups: (**A–C**) the ICU mortality of TBI patients with NLR > 7.44 was higher than those with of NLR ≤ 7.44 at the same sex or the same age phases or the same GCS score level, and (**D–F**) the length of ICU stays of TBI patients with NLR > 7.44 was longer than those with of NLR ≤ 7.44 at the same sex or the same age phases or at the same GCS score level.

As with trauma patients, TBI patients with a high NLR are more likely to receive aggressive care services (ventilation, 44.8% vs. 22.4%, *P* < 0.001; vasopressor, 13.0% vs. 4.0%, *P* < 0.001; antibiotics, 20% vs. 8.6%, *P* < 0.001). Compared with the patients in the low NLR group, patients with the higher NLR had worse clinical outcomes, including ICU and hospital mortality, 15-day mortality, and the length of ICU stay and hospitalization (*P* < 0.001 for all). We compared the ICU mortality of patients with TBI of the same sex or at the same age phases or at the same GCS score level (Figures 3A–C). Patients whose NLR were higher than 7.44 still showed increased ICU mortality. For patients with an NLR ≤ 7.44, the mortalities of male and female patients were 4.3% and 2.1%, respectively, while the mortality rates of male and female patients with an NLR > 7.44 were 13.1% and 7.9%, respectively. The results were similar in the stratified comparison of age and GCS scores. The same analysis applied to length of ICU stay; patients with a high NLR had a longer ICU stay after controlling for sex (Figure 3D), age (Figure 3E), or GCS score (Figure 3F).

#### Multivariate regression model and adjusted survival curves

**Figure 4:**
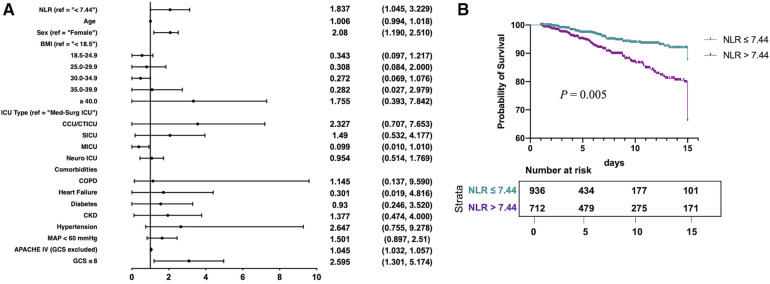
Multivariate regression model and adjusted survival curves in traumatic brain injury patients: (**A**) multivariate logistic regression model was used to show that the higher NLR was an independent risk factor of ICU mortality for traumatic brain injury patients, and (**B**) the adjusted cumulative survival curve indicated the marked survival disadvantage in NLR > 7.44 compared with NLR ≤ 7.44 (*P* = 0.005).

As expected, the multivariate logistic regression model showed a similar trend in the relationship between NLR and ICU mortality risk to that in the univariate analyses (Figure 4A). The increase in the NLR is an independent risk factor leading to ICU death of patients with TBI. The risk of death in the high NLR group was 83.7% higher than that in the group with low NLR. Multivariate adjusted Cox survival curve was also used (Figure 4B), which indicated that compared with patients in the low NLR group, the high NLR group showed an obvious survival advantage. Similar results were obtained when the acquisition time of NLR was extended from 24h to 72h (Supplementary table 3–4).

## Discussion

Our data revealed that the NLR can be used as an index to predict the adverse prognosis of traumatic injury patients, especially those with traumatic brain injury. This may be related to the involvement of lymphocytes and neutrophils in the process of post-traumatic inflammation.

Trauma brings out the release of various inflammatory mediators and causes inflammation (13). In the acute stage of inflammation, various inflammatory cells accumulate and infiltrate to induce Systemic Inflammatory Response Syndrome (SIRS). The further development of inflammatory cells will lead to a multiple organ dysfunction syndrome, which has a very high mortality rate (14). Early detection and active intervention to block the progression of SIRS to MODS are the key factors to reduce the mortality of critically ill patients with traumatic injury. The NLR represents the balance between the inflammatory activator (neutrophil) and the inflammatory regulator (lymphocyte). Manson et al. found that the activation of early lymphocytes was closely related to the development of MODS and the decrease of lymphocyte counts (15). Thus, the more obvious the imbalance of neutrophils and lymphocytes, the more serious the inflammation. A high NLR often indicates poor prognosis, which can be used to indicate and evaluate the severity of the disease. It has been proved that the NLR has a good correlation with the Injury Severity Score (ISS) in trauma patients (16). But the NLR is more objective and accessible than scoring methods, which are highly complicated for practical application and have obvious shortcomings.

It is not difficult to find in this paper that the NLR displays better performance in the traumatic brain injury population than in traumatic patients. GCS, as a common clinical score, is also an independent risk factor for poor prognosis in patients with TBI. However, for patients with intubation, sedation, or severe periorbital swelling, the limitations of the GCS score may be relatively large. Sex also has an impact on the prognosis of trauma patients; the risk of in-hospital death in men is 2.08 times higher than that in females. Many studies have confirmed that acknowledging possible gender differences in a wider range of trauma symptoms may have clinical benefits (17, 18). It is very important to select an easily detected and available laboratory index to further predict the prognosis of patients or improve the accuracy of prognosis prediction. Neutrophils may damage the blood–brain barrier, promote neuron cell death, and induce the expression of oxidase, leading to poor prognosis in TBI patients. The interaction between the brain and the immune system is bidirectional. At the early stage of injury, the levels of catecholamine and steroids increase due to an overactivation of the sympathetic nervous system and hypothalamus pituitary adrenal axis, which induces the apoptosis and dysfunction of peripheral blood lymphocytes (19). Hence, the NLR can reflect the subtle changes of patients' nervous and physical state and make up for the shortcomings of the GCS score. Dynamic monitoring of the changes in the NLR has greater clinical application value.

There are some limitations in this study. First, as this study is a retrospective one, it may lead to a certain degree of bias. The overall AUC of 0.681–0.725 seems not totally enough to be clinically useful in practice. But a prospective observational clinical study will be conducted in our medical center, which is expected to provide stronger evidence in the near future. All data are taken from the period 2014 to 2015. Due to the limited timeliness of the database, more recently admitted ICU patients are not included in the study. We have not been able to provide trauma-related scores such as ISS and Abbreviated Injury Scale (AIS). Also, as a clinical study, we have not been able to fully explain the mechanism behind the NLR. This may require exploration through animal or *in vitro* studies. But this study has its merits. This is a multicenter study based on 208 hospitals. All data are close to real-world observations without human intervention.

## Conclusions

To sum up, the increase in the NLR within 24 h of ICU is associated with a poor prognosis of traumatic patients, especially traumatic brain injury sufferers. An NLR > 7.44 is an independent risk factor of death in patients with TBI. However, this conclusion needs to be further confirmed by prospective clinical research.

## Data Availability

Publicly available datasets were analyzed in this study. This data can be found here: eicu-crd.mit.edu.

## References

[1] Vecino-Ortiz AI Jafri A Hyder AA. Effective interventions for unintentional injuries: a systematic review and mortality impact assessment among the poorest billion. Lancet Glob Health. (2018) 6(5):e523–34. 10.1016/s2214-109x(18)30107-4 29653626

[2] Salehpour F Bazzazi AM Aghazadeh J Hasanloei AV Pasban K Mirzaei F What do you expect from patients with severe head trauma? Asian J Neurosurg. (2018) 13(3):660–3. 10.4103/ajns.AJNS_260_16 30283522 PMC6159042

[3] Hazeldine J Lord JM Belli A. Traumatic brain injury and peripheral immune suppression: primer and prospectus. Front Neurol. (2015) 6:235. 10.3389/fneur.2015.00235 26594196 PMC4633482

[4] Palmer C Roberts RL Young PI: Timing of neutrophil depletion influences long-term neuroprotection in neonatal rat hypoxic-ischemic brain injury. Pediatr Res. (2004) 55(4):549–56. 10.1203/01.Pdr.0000113546.03897.Fc 14739365

[5] Angkananard T Anothaisintawee T McEvoy M Attia J Thakkinstian A. Neutrophil lymphocyte ratio and cardiovascular disease risk: a systematic review and meta-analysis. BioMed Res Int. (2018) 2018:2703518. 10.1155/2018/2703518 30534554 PMC6252240

[6] Wlodarczyk MK Sobolewska AE Stec-Michalska K Fichna JJ Wisniewska-Jarosinska ME. Neutrophil-lymphocyte ratio in Crohn's Disease patients predicts sustained response to infliximab 52-week therapy. J Gastrointestin Liver Dis. (2015) 24(1):127–8. 25822447

[7] Kong W He Y Bao H Zhang W Wang X. Diagnostic value of neutrophil–lymphocyte ratio for predicting the severity of acute pancreatitis: a meta-analysis. Dis Markers. (2020) 2020:9731854. 10.1155/2020/9731854 32454909 PMC7232731

[8] Dilektasli E Inaba K Haltmeier T Wong MD Clark D Benjamin ER The prognostic value of neutrophil-to-lymphocyte ratio on mortality in critically ill trauma patients. J Trauma Acute Care Surg. (2016) 81(5):882–8. 10.1097/ta.0000000000000980 26825931

[9] Zhao JL Du ZY Yuan Q Yu J Sun YR Wu X Prognostic value of neutrophil-to-lymphocyte ratio in predicting the 6-month outcome of patients with traumatic brain injury: a retrospective study. World Neurosurg. (2019) 124:32930–9. 10.1016/j.wneu.2018.12.107 30610986

[10] Pollard TJ Johnson AEW Raffa JD Celi LA Mark RG Badawi O. The eICU collaborative research database, a freely available multi-center database for critical care research. Sci Data. (2018) 5:180178. 10.1038/sdata.2018.178 30204154 PMC6132188

[11] DeLong ER DeLong DM Clarke-Pearson DL. Comparing the areas under two or more correlated receiver operating characteristic curves: a nonparametric approach. Biometrics. (1988) 44(3):837–45. 10.2307/2531595. 3203132

[12] Tolin DF Foa EB. Sex differences in trauma and posttraumatic stress disorder: a quantitative review of 25 years of research. Psychol Bull. (2006) 132(6):959–92. 10.1037/0033-2909.132.6.959 17073529

[13] Lord JM Midwinter MJ Chen YF Belli A Brohi K Kovacs EJ The systemic immune response to trauma: an overview of pathophysiology and treatment. Lancet. (2014) 384(9952):1455–65. 10.1016/s0140-6736(14)60687-5 25390327 PMC4729362

[14] Manson J Thiemermann C Brohi K. Trauma alarmins as activators of damage-induced inflammation. Br J Surg. (2012) 99(Suppl 1):12–20. 10.1002/bjs.7717 22441851

[15] Manson J Cole E De’Ath HD Vulliamy P Meier U Pennington D Early changes within the lymphocyte population are associated with the development of multiple organ dysfunction syndrome in trauma patients. Critical Care. (2016) 20(1):176. 10.1186/s13054-016-1341-2 27268230 PMC4895987

[16] Soulaiman SE Dopa D Raad AT Hasan W Ibrahim N Hasan AY Cohort retrospective study: the neutrophil to lymphocyte ratio as an independent predictor of outcomes at the presentation of the multi-trauma patient. Int J Emerg Med. (2020) 13(1):5. 10.1186/s12245-020-0266-3 32019485 PMC7001256

[17] Kondo Y Miyazato A Okamoto K Tanaka H. Impact of sex differences on mortality in patients with sepsis after trauma: a nationwide cohort study. Front Immunol. (2021) 12:678156. 10.3389/fimmu.2021.678156 34267751 PMC8276106

[18] Kwan RO Cureton EL Dozier KC Victorino GP: Gender differences among recidivist trauma patients. J Surg Res. (2011) 165(1):25–9. 10.1016/j.jss.2010.05.060 20828752

[19] Meisel C Schwab JM Prass K Meisel A Dirnagl U: Central nervous system injury-induced immune deficiency syndrome. Nat Rev Neurosci. (2005) 6(10):775–86. 10.1038/nrn1765 16163382

