# Supplementary material for: The neutrophil-to-lymphocyte ratio: A potential predictor of poor prognosis in adult patients with trauma and traumatic brain injury
Source: Front Surg. 2022 Aug 23;9:917172. doi: 10.3389/fsurg.2022.917172 (PMC9445209; doi:10.3389/fsurg.2022.917172)
Supplement: Supplementary file 1 [file Table_1_v1.docx]

Supplementary table 1. Univariate analysis of clinical outcomes in 5157 patients by NLR category

|  | Group 1 | Group 2 | Group 3 | Group 4 | *P* |
| --- | --- | --- | --- | --- | --- |
|  | ＜5.00 (1301) | 5.00-8.40 (1281) | 8.40-14.33 (1278) | ＞14.33 (1297) |  |
| Ventilation n (%) | 198 (15.2) ^a^ | 358 (27.9) ^b^ | 474 (37.1) ^c^ | 581 (44.8) ^d^ | ＜0.001 |
| Dialysis n (%) | 13 (1.0) ^a^ | 22 (1.7) ^a^ | 27 (2.1) ^a^ | 46 (3.5) ^b^ | ＜0.001 |
| Vasopressor n (%) | 41 (3.2) ^a^ | 87 (6.8) ^a^ | 126 (9.9) ^b^ | 220 (17.0) ^c^ | ＜0.001 |
| Antibiotics n (%) | 157 (12.1) ^a^ | 208 (16.2) ^b^ | 264 (20.7) ^c^ | 377 (29.1) ^d^ | ＜0.001 |
| ICU LOS (days) | 1.50 (0.92-2.54) | 2.08 (1.27-4.12) | 2.79(1.54-5.80) | 3.54 (1.70-6.91) | ＜0.001 |
| Hospital LOS (days) | 4.33 (2.50-7.69) | 6.00 (3.69-10.60) | 7.75 (4.67-12.97) | 8.21 (4.91-14.98) | ＜0.001 |
| ICU mortality, n (%) | 17 (1.3) ^a^ | 44 (3.4) ^b^ | 73 (5.7) ^c^ | 153 (11.1) ^d^ | ＜0.001 |
| In-Hospital mortality, n (%) | 25 (1.9) a | 67 (5.3) b | 113 (9.0) ^b^ | 231 (18.1) ^c^ | ＜0.001 |

^a, b, c, d^: The *P* value of the two groups marked with the same letter was greater than the adjusted *P* value, and there was no statistical difference; otherwise, the *P* value of the two groups marked with the different letter is smaller than the adjusted *P* value, there was statistical difference.

LOS: length of stay.

Supplementary table 2. Multivariate regression model in traumatic patients

| Covariant | P | Exp (B) | 95%CI | |
| --- | --- | --- | --- | --- |
|  |  |  | Lower | Upper |
| NLR (ref = “< 5.00”) |  |  |  |  |
| 5.00-8.40 | 0.166 | 1.629 | 0.816 | 3.249 |
| 8.40-14.33 | 0.009 | 2.363 | 1.242 | 4.494 |
| ＞14.33 | ＜0.0001 | 3.499 | 1.883 | 6.503 |
| Sex (ref = “Female”) | 0.057 | 1.377 | 0.991 | 1.914 |
| Age | 0.549 | 0.998 | 0.990 | 1.005 |
| APACHE IV | ＜0.0001 | 1.054 | 1.048 | 1.060 |
| BMI (ref = “< 18.5”) |  |  |  |  |
| 18.5-24.9 | 0.473 | 0.782 | 0.400 | 1.530 |
| 25.0-29.9 | 0.044 | 0.488 | 0.243 | 0.980 |
| 30.0-34.9 | 0.204 | 0.621 | 0.298 | 1.295 |
| 35.0-39.9 | 0.929 | 1.038 | 0.456 | 2.361 |
| ≥40.0 | 0.430 | 0.688 | 0.273 | 1.738 |
| Comorbidities |  |  |  |  |
| Hypertension | 0.895 | 0.970 | 0.614 | 1.532 |
| Diabetes | 0.065 | 0.563 | 0.306 | 1.036 |
| CKD | 0.042 | 1.729 | 1.021 | 2.929 |
| COPD | 0.450 | 0.762 | 0.376 | 1.544 |
| Heart Failure | 0.177 | 1.579 | 0.814 | 3.064 |

Supplementary table 3. Univariate analysis of clinical outcomes in 2284 patients with TBI by NLR category

|  | Low | High | *P* |
| --- | --- | --- | --- |
|  | NLR ≤ 7.45 (1015) | NLR > 7.45 (1269) |  |
| Ventilation, n (%) | 222 (21.6) | 526 (40.8) | ＜0.001 |
| Dialysis, n (%) | 8 (0.8) | 29 (2.2) | 0.005 |
| Vasopressor n (%) | 44 (4.3) | 138 (10.7) | ＜0.001 |
| Antibiotics n (%) | 94 (9.1) | 203 (15.7) | ＜0.001 |
| ICU LOS (days) | 1.67 (0.96-2.96) | 2.83 (1.54-5.79) | 0.004 |
| Hospital LOS (days) | 4.88 (2.79-8.38) | 7.90 (4.83-13.32) | 0.034 |
| ICU mortality, n (%) | 23 (2.2) | 101 (7.8) | ＜0.001 |
| 15-day mortality, n (%) | 32 (3.1) | 137 (10.7) | ＜0.001 |
| In-Hospital mortality, n (%) | 34 (3.3) | 157 (12.4) | ＜0.001 |

Supplementary table 4. Multivariate regression model in traumatic brain injury patients

| Covariant | P | Exp (B) | 95%CI | |
| --- | --- | --- | --- | --- |
|  |  |  | Lower | Upper |
| NLR (ref = “< 7.44”) | ＜0.0001 | 3.789 | 1.949 | 7.369 |
| Age | 0.491 | 1.004 | 0.992 | 1.017 |
| Sex (ref = “Female”) | 0.004 | 2.425 | 1.320 | 4.457 |
| BMI (ref = “< 18.5”) |  |  |  |  |
| 18.5-24.9 | 0.824 | 1.186 | 0.263 | 5.340 |
| 25.0-29.9 | 0.973 | 1.026 | 0.222 | 4.750 |
| 30.0-34.9 | 0.423 | 1.874 | 0.403 | 8.727 |
| 35.0-39.9 | 0.338 | 2.238 | 0.430 | 11.636 |
| ≥40.0 | 0.997 | 0.000 | 0.000 | - |
| ICU Type (ref = “Med-Surg ICU”) |  |  |  |  |
| CCU/CTICU | 0.274 | 0.319 | 0.041 | 2.476 |
| SICU | 0.462 | 0.669 | 0.228 | 1.958 |
| MICU | 0.998 | 0.000 | 0.000 | - |
| Neuro ICU | 0.972 | 0.989 | 0.540 | 1.812 |
| Comorbidities |  |  |  |  |
| COPD | 0.794 | 0.750 | 0.086 | 6.522 |
| Heart Failure | 0.998 | 0.000 | 0.000 | - |
| Diabetes | 0.445 | 0.440 | 0.053 | 3.623 |
| CKD | 0.149 | 2.391 | 0.732 | 7.808 |
| Hypertension | 0.549 | 1.3555 | 0.502 | 3.658 |
| MAP＜60mmHg | 0.801 | 1.998 | 0.000 | - |
| APACHE IV (GCS excluded) | ＜0.0001 | 1.111 | 1.012 | 1.218 |
| GCS≤8 | ＜0.0001 | 2.801 | 1.239 | 5.924 |
